# WNT2-Mediated FZD2 Stabilization Regulates Esophageal Cancer Metastasis via STAT3 Signaling

**DOI:** 10.3389/fonc.2020.01168

**Published:** 2020-07-16

**Authors:** Yufei Fu, Qi Zheng, Yingying Mao, Xiyi Jiang, Xin Chen, Pei Liu, Bin Lv, Tuxiong Huang, Jiao Yang, Yongran Cheng, Xiaoyi Dai, Chunyan Dai, Xi Wang, Yifei Yin, Tengjiao Song, Weiyang Jin, Chang Zou, Tianhui Chen, Li Fu, Zhe Chen

**Affiliations:** ^1^Key Laboratory of Digestive Pathophysiology of Zhejiang Province, First Affiliated Hospital, Zhejiang Chinese Medical University, Hangzhou, China; ^2^Department of Thoracic and Cardiovascular Surgery, The First Affiliated Hospital of Zhejiang University, Hangzhou, China; ^3^Department of Epidemiology and Biostatistics, Zhejiang Chinese Medical University, Hangzhou, China; ^4^Group of Molecular Epidemiology & Cancer Precision Prevention, Zhejiang Academy of Medical Sciences, Hangzhou, China; ^5^Guangdong Provincial Key Laboratory of Regional Immunity and Diseases, Department of Pharmacology and Shenzhen International Cancer Centre, Shenzhen University School of Medicine, Shenzhen, China; ^6^College of Life Science, Zhejiang Chinese Medical University, Hangzhou, China; ^7^College of Life and Environmental Sciences, Hangzhou Normal University, Hangzhou, China; ^8^Clinical Medical Research Center, The First Affiliated Hospital of Southern University of Science and Technology, The Second Clinical Medical College of Jinan University, Shenzhen People's Hospital, Shenzhen, China; ^9^Department of Cancer Prevention, Cancer Hospital of the University of Chinese Academy of Sciences (Zhejiang Cancer Hospital), Hangzhou, China; ^10^Institute of Cancer and Basic Medicine, Chinese Academy of Sciences, Hangzhou, China

**Keywords:** esophageal squamous cell carcinoma (ESCC), metastasis, WNT2, FZD2, ubiquitination, STAT3signaling

## Abstract

Esophageal cancer micro environment factor WNT2 was critical in cancer metastasis. However, very little is known about WNT2 receptors and their role in the malignant progression of ESCC. The clinical significance and underlying molecular mechanisms of FZD2, one of the receptors of WNT2, was further investigated in ESCC. We found that FZD2 expression was positively correlated with WNT2 levels in clinical ESCC specimens through database analysis. Upregulated FZD2 expression was detected in 69% (69/100) of the primary ESCC cases examined, and increased FZD2 expression was significantly correlated with poor prognosis (*P* < 0.05). Mechanistically, FZD2 induced the migration and invasion of ESCC cells by regulating the FZD2/STAT3 signaling. *In vivo* xenograft experiments further revealed the metastasis-promoting role of FZD2 in ESCC. Moreover, we found that the WNT2 ligand could stabilize and phosphorylate the FZD2 receptor by attenuating FZD2 ubiquitination, leading to the activation of STAT3 signaling and the initiation of ESCC cell metastasis. Collectively, our data revealed that a novel non-canonical WNT2/FZD2/STAT3 signaling axis is critical for ESCC progression. Strategies targeting this specific signaling axis might be developed to treat patients with ESCC.

## Introduction

Esophageal carcinoma (EC) is the sixth leading cause of cancer-related death worldwide due to its high risk of metastasis and recurrence (1). The most prevalent histological subtype of EC in eastern Asia (particularly in China) and in eastern and southern Africa is esophageal squamous cell carcinoma (ESCC) (2, 3). The 5-year post-operative survival rate of ESCC patients remains below 30% because a large proportion of these patients are diagnosed at an advanced stage with distant metastasis (4). Lymph node (LN) metastasis is an indicator of distant metastasis and poor prognosis for patients with ESCC (5). Metastasis to regional LNs is a complex process. Aberrant WNT signaling, one of the key signaling cascades that regulates the tumourigenesis and metastasis in many types of cancers, is also observed in ESCC (6, 7). However, the mechanisms by which the WNT pathway is activated during ESCC metastasis remain to be further explored.

WNT ligands have been reported to bind to a panel of different receptors to preferentially activate the canonical and/or non-canonical WNT signaling, including ten transmembrane receptors of the frizzled (FZD) family (8). The WNT/FZD axis has been found to be a critical mediator of tumor-stromal interactions, promoting the metastasis of breast, colon, stomach, thyroid, and lung cancer cells (9–13). In addition, FZD2 is up-regulated in many types of cancers, which is considered to be a new predictor of tumor recurrence. It is involved in the metastasis of various cancers including endometrial cancer (14), neuroblastoma (15), oral squamous cell carcinoma (16), tongue squamous cell carcinoma (17), hepatocellular carcinoma (18), colon cancer (19), and prostate cancer (20, 21). Recently, the down-regulation of miR-30a-3p/5p was reported to promote ESCC cell proliferation through the up-regulation of WNT2 and FZD2, respectively (7). Furthermore, WNT2, which is also known to be a robust factor that mediates tumor-stromal interactions, is one of the up-regulated WNT genes in cancer-associated fibroblasts isolated from primary ESCC, which may contribute to the invasiveness of ESCC cells (22). In our present study, FZD2 was found to be a WNT2 receptor. However, the functions and mechanisms of WNT2-FZD2 in the progression of ESCC remain unclear.

FZD2 activates both the β-catenin-dependent (canonical) and β-catenin-independent (non-canonical) signaling (23). For instance, it has been demonstrated that in the presence of WNT3a, FZD2 activates β-catenin-dependent signaling in lung cancer (24), while in melanoma cell lines, WNT5a/FZD2 signaling activates the non-canonical WNT/Ca^2+^ pathway (25). In addition, in metastatic liver, lung, colon and breast cancer cell lines, FZD2 drives the epithelial-mesenchymal transition (EMT) and cell migration through the non-canonical WNT5a/FZD2/STAT3 pathway (26).

In the present study, we aimed to investigate the interaction between WNT2 and FZD2, as well as the molecular mechanisms of the WNT2-FZD2 signaling axis in the progression of ESCC.

## Materials and Methods

### Analysis of ESCC Data From the Cancer Genome Atlas (TCGA) and Gene Expression Omnibus (GEO) Databases

The ESCC dataset was downloaded from the TCGA portal (https://tcga-data.nci.nih.gov/docs/publications/tcga) (27). The dataset included the RNA expression levels in 85 tumor tissues and 3 matched non-tumor tissues, generated using Illumina sequencing technology. Another two datasets [GSE20347 (28) and GSE77861 (29)] from the GEO database (https://www.ncbi.nlm.nih.gov/geo/) (30) were also downloaded. Paired *t*-tests were used to analyse the differences between ESCC tumor and normal tissues. Correlations between the expression of FZD2 and EMT-related genes (26) from the TCGA database were assessed using Pearson's correlation analysis; adjusted *P*-values (rounded to three decimal places) were calculated using the FDR method. An adjusted *P* < 0.05 was considered as statistically significant.

### Tissue Collection

In total, 100 primary ESCC tissues and 80 corresponding adjacent non-tumor tissues collected from the National Human Genetic Resources Sharing Service Platform (No. 2005DKA21300) were used as tissue array samples. Additionally, 8 other pairs of tumor and normal tissues were collected from the First Affiliated Hospital of Zhejiang University (Hangzhou, China). Written informed consent for the use of the collected samples was obtained from all participants. This study was approved by the Institutional Review Board and Ethics Committee of the First Affiliated Hospital of Zhejiang University.

### Immunohistochemical (IHC) Staining

IHC staining was performed to detect FZD2 expression in tissue samples. The standard streptavidin–biotin–peroxidase complex method was used for IHC staining. Briefly, after blocking of endogenous peroxidase activity in tissue sections with 3% H_2_O_2_ and antigen retrieval with a target retrieval solution (S1699; Agilent Technologies Inc., Santa Clara, CA, USA) according to the manufacturer's instructions, the sections were incubated with 10% normal goat serum (S-1000; Vector Laboratories, Burlingame, CA, USA) in PBS for 30 min. Next, sections were incubated with a primary anti-FZD2 antibody (ab109094, 1:200, Abcam, Cambridge, UK) at 4°C overnight. After three washes with PBS, the sections were incubated with donkey anti-goat IgG H&L (HRP) (ab205723, 1:2000, Abcam, Cambridge, UK) for 30 min at room temperature. Finally, sections were incubated with a peroxidase substrate solution (Sk-4100, Vector Laboratories, Burlingame, CA, USA) until the desired staining intensity was attained. Sections were rinsed with tap water, counterstained with haematoxylin, and mounted with coverslips. The results of IHC staining were viewed and scored separately by two experienced pathologists. The expression levels of FZD2 expression were assessed and scored as follows: negative (0; complete absence of staining), weak staining (score: 1), moderate staining (score: 2), or strong staining (score: 3).

### Cell Culture and Reagents

HEK293T cells and the human ESCC cell line KYSE150 were obtained from the Cell Bank of Chinese Academy of Sciences (Shanghai, P. R. China). The human ESCC cell lines KYSE30 and KYSE410 were obtained from the China Centre for Type Culture Collection (Wuhan, P. R. China). All the cells were verified using short tandem repeats (STRs). All experiments were performed with mycoplasma-free cells. HEK293T and KYSE30 cells were maintained in DMEM (Thermo Fisher Scientific, Waltham, MA, USA) supplemented with 10% fetal bovine serum (FBS; Thermo Fisher Scientific, Waltham, MA, USA). KYSE150 and KYSE410 cells were cultured in RPMI-1640 medium (Thermo Fisher Scientific) supplemented with 10% FBS. Cells were incubated at 37°C in a 5% CO_2_ atmosphere. The recombinant human WNT2 protein was purchased from Mulder Company (Hangzhou, P. R. China). All cells were cultured in the biosafety level 2 laboratory.

### RNA Extraction, Reverse Transcription, and Quantitative Real-Time PCR

Total RNA was extracted from cells using TRIzol (Thermo Fisher Scientific). Reverse transcription was performed using the PrimeScript™ II 1st Strand cDNA Synthesis Kit from Takara (Beijing, P.R. China) according to the manufacturer's instructions. Amplification by real-time PCR was performed using Luna Universal qPCR Master Mix (New England Biolabs, UK) according to the manufacturer's protocol. The following primer sequences were used for qPCR: FZD2, forward 5′- GTGCCATCCTATCTCAGCTACA-3′ and reverse 5′-CTGCATGTCTACCAAGT ACGTG-3′; β-Actin, forward 5′-CATGTACGTTGCTATCCAGGC-3′ and reverse 5′-CTCCTTAATGTCACGCACGAT-3′. Cycle threshold (Ct) values were calculated, and the relative mRNA levels of targeted genes were analyzed using the 2^−ΔΔ*CT*^ method.

### Western Blot Analysis

Cells were harvested, washed and lysed in lysis buffer supplemented with a protease/phosphatase inhibitor cocktail (Cell Signaling Technology, MA, USA). Proteins were separated using SDS-PAGE and transferred to PVDF membranes (Millipore, Bedford, MA, USA). Membranes were blocked with 5% skim milk in tris buffered saline containing 0.5% Tween-20 (TBST) overnight and then incubated with primary antibodies at 4°C. The primary antibodies were against FZD2 (1:1,000 dilution, R&D Systems, Minneapolis, MN, USA), TWIST1 (1:1,000 dilution, R&D Systems), Slug, STAT3, p-STAT3-Tyr705, p-STAT3-Ser727, active β-catenin, β-catenin, HA tag, GAPDH, Mcl-1, Bcl-2, cIAP-2, and survivin (1:1,000 dilution, Cell Signaling Technology), Flag-tag and β-Actin (1:3,000 dilution, Sigma-Aldrich, Merck KGaA, St Louis, MO, USA), Cyclin D1 (1:5,000 dilution, Abcam), and Ub (1:500, Santa Cruz). Membranes were then incubated with secondary antibodies (1:5,000 dilution, Lianke Bio, P.R. China). Proteins were detected using an Enhanced Chemiluminescence Kit (Millipore) according to the manufacturer's instructions. An anti-β-actin antibody (Sigma-Aldrich) was used to detect uniform loading.

### Immunofluorescence Staining

Cells were grown to 80% confluence on glass coverslips. Then, the cell layers were washed with PBS and fixed with 4% paraformaldehyde (PFA). Cells were incubated with ice-cold 100% methanol for 10 min at −20°C. After rinsing with PBS for 5 min, the cells were incubated with blocking buffer (1 × PBS/5% normal serum/0.3% Triton™ X-100) for 60 min. The anti-β-catenin antibody (1:100 dilution, Cell Signaling Technology) was diluted in antibody dilution buffer (1 × PBS/1% albumin from bovine serum albumin (BSA)/0.3% Triton™ X-100). Cells were incubated with diluted primary antibodies overnight at 4°C. After thorough washing, cells were incubated with fluorescent dye-conjugated secondary antibodies (1:1,000 dilution, Cell Signaling Technology) for 1 h at room temperature. Finally, cells were washed with PBS and stained with DAPI (Thermo Fisher Scientific) for 10 min at room temperature in the dark. Slides were covered with mounting medium, and images were captured under a Leica TCS SP8 fluorescence confocal microscope (Wetzlar, Germany).

### Cell Counting Kit-8 (CCK-8) Assay

Tumor cell growth was quantified using the CCK-8 assay (Dojindo, P.R. China) according to the manufacturer's instructions. Briefly, cells were plated in 96-well plates (3 × 10^4^ cells/well) and incubated with 100 μL of medium overnight. The absorbance at 450 nm using a microplate spectrophotometer (Varioskan Flash, Thermo Fisher Scientific) after culturing the cells with 10 μL of CCK-8 reagent. The *P*-values and *F*-values were carried out by GraphPad Prism with two-way ANOVA analysis.

### Lentiviral Packaging and Infection

Lentiviral vectors encoding the human FZD2 shRNA were designed and synthesized by GeneChem (Shanghai, P.R. China). Transfection was performed according to the manufacturer's instructions. The RNAi sequences targeting FZD2 gene were 5′-CCACGTACTTGGTAGACAT-3′ and the negative control sequence was 5′-TTCTCCGAACGTGTCACGT-3′. The knockdown efficiency was evaluated using fluorescence microscopy, qPCR and western blot analysis. The lentiviral vectors overexpressing FZD2 (pGC-FU-3FLAG-SV40-EGFP-IRES-Puromycin) and the empty lentiviral vector for control were purchased from GeneChem (Shanghai, P.R. China). Both the lentiviral vector overexpressed WNT2 (pCDH-CMV-MCS-EF1-RFP) and the empty lentiviral vector for control were purchased from Mulder Company (Hangzhou, P. R. China). Stably transfected cells were selected according to the manufacturer's instructions. The overexpression of FZD2 was verified using qPCR, western blot analysis and fluorescence microscopy.

### Wound-Healing Assay

Wound-healing assays were performed using 12-well plates. Cells were grown to a confluent monolayer for 24 h before the assay. The surface of the monolayer was carefully scratched with a 200 μL sterile pipette tip. The wells were washed twice with fresh medium. Cells were imaged under a light microscope at 0, 8, and 16 h after scratch. For each sample, an image of the scratched area was captured at least three times.

### Cell Migration and Invasion Assays

Cells were cultured in serum-free medium for 24 h before performing the migration and invasion assays. Cells (1 × 10^5^) in 0.5 mL of serum-free medium were seeded in the upper chamber (8 μm pore size, BD Biosciences, San Jose, CA, USA) with 40 μL of 1 mg/mL Matrigel, and 0.7 mL of complete medium containing 10% FBS was added to the lower chamber. After a 48-h incubation, the cells on the top of the membrane (non-migrating) were removed with cotton swabs. Cells that had migrated to the bottom well were fixed with methanol for 10 mins and stained with a 0.05% crystal violet solution. The number of invading cells was counted in three randomly selected light microscopy fields (Olympus Corporation, Japan, magnification, 40 ×).

### Co-immunoprecipitation and Ubiquitination Assay

Cells were cultured in 6-well plates at a density of 5 × 10^5^ cells/well overnight before being transfected with the indicated plasmids. After 36 h, the cells were incubated with 20 μM MG132 for 4 h. Total proteins were extracted using cell lysis buffer containing a protease/phosphatase inhibitor cocktail (Cell Signaling Technology). Cell lysates were incubated with anti-Flag M2 affinity gel (A2220, Sigma-Aldrich) at 4°C overnight. After washing the beads three times with cell lysis buffer, the immunoprecipitates were collected for immunoblotting. For HA-tagged protein and Ubiquitin protein immunoprecipitation, the lysates were incubated with 1 mg of an anti-HA (3,724, Cell Signaling Technology) antibody, an anti-ubiquitin antibody (sc-271289, Santa Cruz) or IgG overnight and were then incubated with 30 μL of 50% GammaBind plus Sepharose beads (17-0886-01, GE Healthcare, Sweden) for an additional 4 h. After washing the beads three times with cold lysis buffer, the immunoprecipitated proteins were analyzed using immunoblotting.

### *In vivo* LN Metastasis Assay

The study protocol was approved by the Experimental Animals Ethics at the Zhejiang Chinese Medical University. The study was performed as previously described (31). KYSE150-negative control (NC) and KYSE150-shFZD2 cells (2 × 10^5^ mean cells) were subcutaneously injected into the foot pads of 4-week-old male SCID mice (*n* = 5). Gentle pressure was applied to the mouse's hind limb above the foot during the injection to prevent the overflow of cell suspension out of the foot pad and up to the leg. The mice were examined for tumor growth in the foot pads at 15-day post-injection. Live imaging of luciferase bioluminescence was performed every 5 days for 1 month beginning at 15-day post-injection. Following sacrifice, swollen inguinal LNs, metastatic tumor cells and tumors were collected, fixed with 4% PFA and embedded in paraffin blocks for haematoxylin and eosin (H&E) staining.

### Statistical Analysis

The expression levels of FZD2 in ESCC and normal tissues are shown as the mean ± SD, and differences in expression levels between the tumor and normal tissues were analyzed using the paired *t-*test. The correlation between FZD2 expression and clinic-pathological features was assessed by calculating Spearman's correlation coefficient, and *P-*values were evaluated using two-tailed *t-*tests. All statistical analyses were performed using SPSS (PASW Statistics 18, IBM, USA) software. A two-sided *P* < 0.05 was considered statistically significant.

## Results

### Overexpression of FZD2 in Primary ESCC Correlates With WNT2 Expression, EMT, and a Poor Prognosis

**Figure 1:**
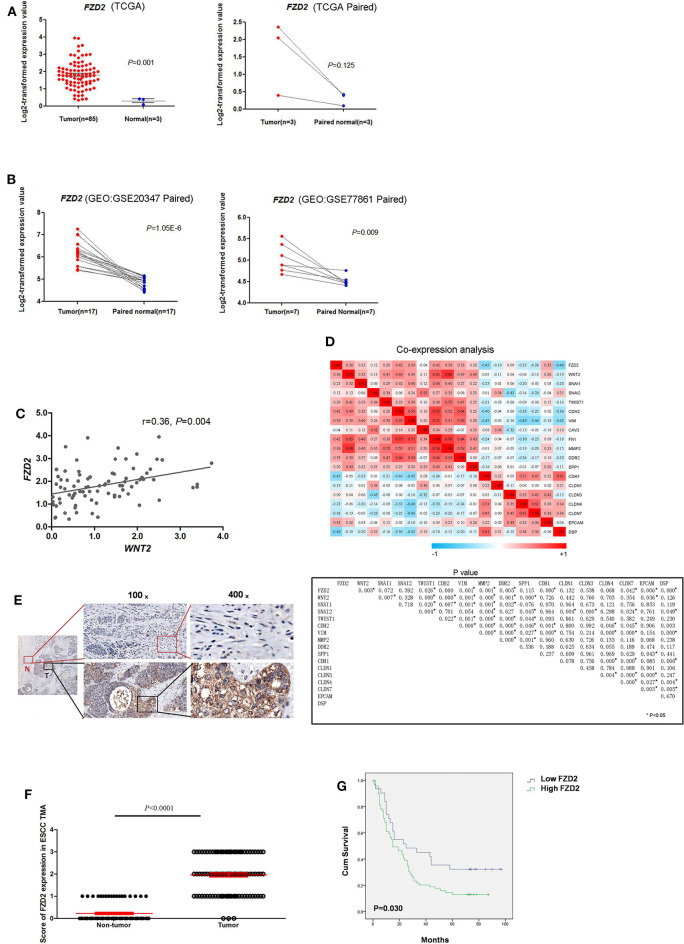
FZD2 was significantly up-regulated in patients with ESCC. The expression levels of FZD2 in non-matched and matched ESCC and normal tissue datasets from the TCGA **(A)** and GEO **(B)** databases were analyzed. **(C)** The correlation between WNT2 and FZD2 in ESCC and normal tissue datasets from the TCGA database. **(D)** Heatmaps showed the correlations between FZD2 and WNT2 expression with the mRNA expression of mesenchymal and epithelial markers in ESCC tissues in datasets from the TCGA. *P*-values were adjusted using the FDR method and are shown below. **(E)** IHC staining showed higher FZD2 expression in ESCC tissues (T) and lower or negative expression in adjacent non-tumor tissues (N). **(F)** Scores corresponding to the FZD2 expression levels in non-tumor and ESCC tissues from all 180 informative cases from the TMA. **(G)** Kaplan-Meier survival analysis stratified according to the FZD2 expression in 100 informative ESCC samples (*P* = 0.030 using the log-rank test) (*P < 0.05).

The levels of *FZD2* mRNA in ESCC and normal tissues were first analyzed using public databases. In the TCGA database, significantly higher levels of *FZD2* mRNA were observed in the ESCC tissues than in the normal tissues (1.80 ± 0.78 vs. 0.30 ± 0.18, *P* = 0.001) (Figure 1A). Two additional datasets (GSE20347 and GSE77861) containing ESCC tissues from the GEO database were also used. In both datasets, the *FZD2* mRNA was expressed at higher levels in the ESCC tissues than in the normal tissues (Figure 1B).

Our previous study showed that CAF-secreted WNT2 is a critical tumor microenvironment factor that can enhance ESCC cell motility and invasiveness (22). Thus, we initially analyzed the correlation of *WNT2* and its potential receptor *FZD2* at the mRNA level in ESCC using the TCGA database. The mRNA levels of *FZD2* and *WNT2* were positively correlated in primary ESCCs (*r* = 0.36, *P* = 0.004) (Figure 1C).

Moreover, the mRNA levels of FZD2 were positively correlated with mesenchymal cell markers, such as Snail (*SNAI1*), Slug (*SNAI2*), Vimentin (*VIM*), N-cadherin (*CDH2*), and Fibronectin (*FN1*) but were negatively correlated with epithelial cell markers such as Epcam (*Epcam*) and E-cadherin (*CDH1*) (Figure 1D). Based on these results, the expression of *FZD2* mRNA was up-regulated in ESCC tissues and positively correlated with the expression of *WNT2* and EMT-related genes.

Next, IHC staining of tumor and normal tissues from 8 ESCC patients were performed. The results showed that FZD2 was heterogeneouslly overexpressed in tumor tissues and weakly expressed in non-tumor tissues (Figure 1E). Then, a tissue microarray (TMA) was constructed and subjected to IHC staining to examine the levels of FZD2 protein in 100 primary ESCC tissues and 80 corresponding adjacent normal tissues (Table 1). A double-blinded evaluation of the IHC staining results was performed according to the clinico-pathological standard (Table 1). As shown in Table 1, moderate-to-strong membrane staining of FZD2 was detected in 69% (69/100) of the ESCC tissues, whereas negative-to-weak membrane staining of FZD2 was observed in 31% (31/100) of the ESCC tissues. The FZD2 protein level was significantly higher in the ESCC tissues than in the normal tissues (*P* < 0.0001; Figure 1F and Table 2). Furthermore, the Kaplan-Meier analysis results revealed a shorter survival time for patients with ESCC who exhibited high FZD2 expression (*n* = 69, median survival time = 27 months) than those who exhibited low FZD2 expression (*N* = 31, median survival time = 45 months; *P* = 0.030; Figure 1G). The correlations between FZD2 expression and the clinico-pathological features of ESCC patients were also analyzed. High expression of FZD2 protein was positively correlated with more progressive pathological grade (*P* < 0.05) and more advanced clinical stage (*P* < 0.05). However, high FZD2 expression was negatively correlated with tumor size (*P* < 0.05) (Table S1). Thus, FZD2 was expressed at high levels in ESCC tissues and was associated with a poor prognosis.

**Table 1:** Demographic and clinicopathological features of ESCC patients *(n* = 100).

**Variables**	**N (%)**
**Age (years)**	
<= 60	32 (32%)
>60	68 (68%)
**Sex**	
Male	74 (74%)
Female	26 (26%)
**Tumor size**	
<= 5 cm	57 (57%)
>5 cm	28 (28%)
Unknown	15 (15%)
**Pathological grade**	
I	6 (6%)
I–II, II	66 (66%)
II–III, III	28 (28%)
**Clinical stage**	
1	4 (4%)
2	42 (42%)
3	50 (50%)
**Tumor stage (T)**	
I	4 (4%)
II	11 (11%)
III	79 (79%)
IV	3 (3%)
**Lymph node metastases (N)**	
N0	45 (45%)
N1	31 (31%)
N2	17 (17%)
N3	5 (5%)
Nx	1 (1%)
Unknown	1 (1%)
**Distance metastases (M)**	
M0	99 (99%)
M1	0
Unknown	1 (1.11%)
**FZD2 staining intensity**	
0, 1	31 (31%)
2, 3	69 (69%)

**Table 2:** Expression of FZD2 in ESCC and normal tissues.

**Sample**	**Number**	Expressions of FZD2*	***P*-value**
ESCC tumor tissues	80	1.89 ± 0.42	0.000***
Normal tissues	80	0.23 ± 0.86	

* *Expressions of FZD2 in ESCC were shown as mean ± SD*.

*** *P < 0.001*.

### FZD2 Promotes the Migration and Invasion of Human ESCC Cells

**Figure 2:**
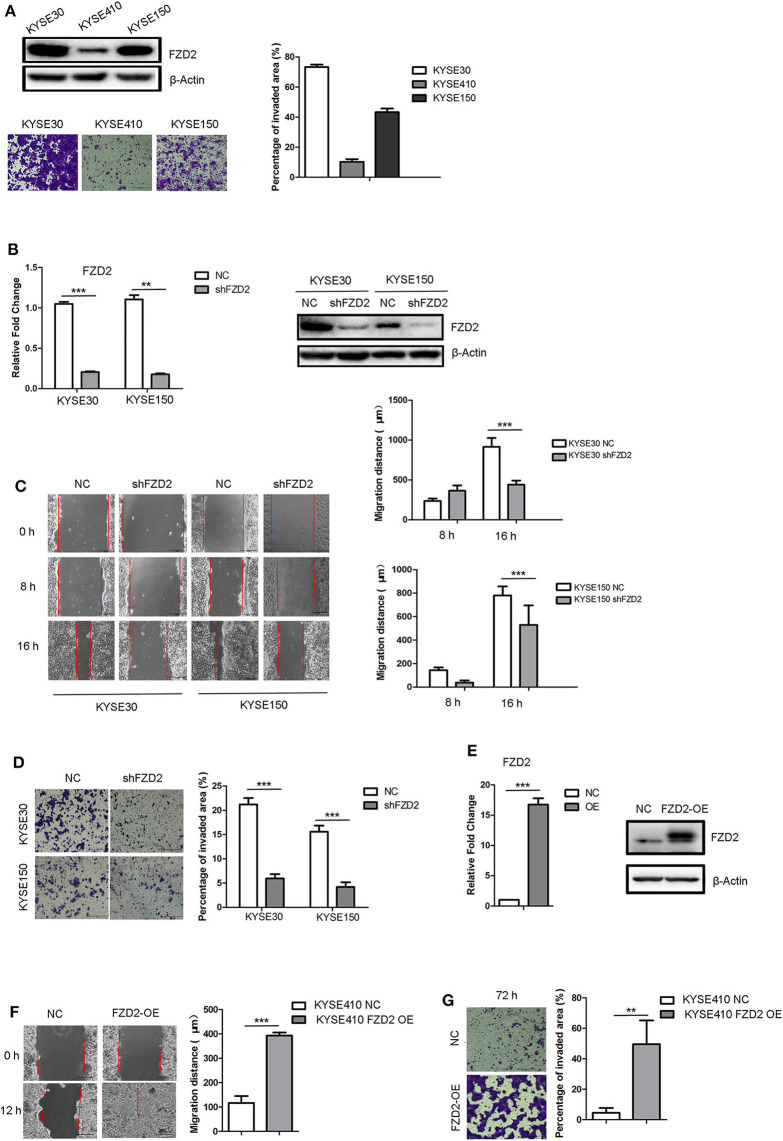
FZD2 knockdown inhibited tumor migration and invasion, while FZD2 overexpression promoted tumor migration and invasion *in vitro*. **(A)** The expression levels of FZD2 in ESCC cell lines were assessed. Representative images of staining showed the different invasive capacities of ESCC cell lines, and the percentages of the invaded area were quantified. **(B)** The expression levels of FZD2 in FZD2-knockdown cell lines were examined using qPCR and western blot analyses. **(C)** Decreased migration and **(D)** invasion of FZD2-knockdown cells was observed compared to the control cells. Average migration distances and the percentages of invaded area are presented as the mean ± SD of triplicate experiments (***P < 0.001 using *t*-test). **(E)** The expression levels of FZD2 in FZD2-overexpressing cell lines were determined using qPCR and western blot analysis. **(F)** Increased migration and **(G)** invasion of FZD2-overexpressing cells were observed compared with the control cells. Average migration distances and invaded area percentages are presented as the mean ± SD of triplicate experiments (**P < 0.001 and ***P < 0.001 using *t*-test).

We examined the expression levels of FZD2 in three ESCC cell lines (KYSE30, KYSE410, and KYSE150) to investigate the functional consequences of high FZD2 expression in ESCC. Higher expression of FZD2 was detected in KYSE30 and KYSE150 cells than in the KYSE410 cells (Figure 2A). Transwell invasion assays were then performed to assess the correlation between FZD2 levels and the invasive capacity of these ESCC cell lines. The levels of FZD2 expression positively correlated with the invasive capacity of ESCC cells (Figure 2A).

Next, stable lines of KYSE30 and KYSE150 cells with lentivirus-mediated knockdown of FZD2 expression (KYSE30/KYSE150-shFZD2) were established. Non-template shRNA-transfected cells (KYSE30/KYSE150-NC) were also established as controls. The knockdown efficiency of FZD2 was confirmed using qPCR and western blot analysis (Figure 2B). Migration and invasion assays were subsequently conducted and the results showed that the migration (Figure 2C) and invasion (Figure 2D) of KYSE30-shFZD2 and KYSE150-shFZD2 were suppressed compared with those in the KYSE30-NC and KYSE150-NC cells, respectively.

We established stable FZD2-overexpressing (FZD2-OE) KYSE410 cells and vector-transfected KYSE410 cells as controls (NC) to substantiate the findings described above. Both qPCR and western blot analyses were used to confirm the FZD2 overexpression in KYSE410 cells (Figure 2E). FZD2-OE KYSE410 and control cells were used to perform migration and invasion assays. Compared with the control cells, FZD2 overexpression promoted cell migration and invasion (Figures 2F,G). These findings support the hypothesis that FZD2 is a key factor in modulating ESCC metastasis.

### FZD2/STAT3 Signaling Induces TWIST1 and Slug Expression in ESCC Cells

**Figure 3:**
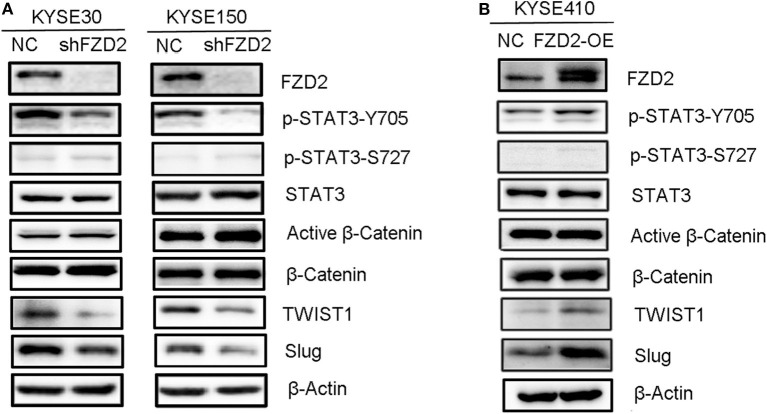
FZD2-induced expression of TWIST1 and Slug in ESCC cells was dependent on STAT3 activation but not β-catenin activation. Western blot analysis of FZD2-knockdown cells **(A)**, FZD2-overexpressing cells **(B)**, and corresponding control cells.

Here, we confirmed that the migration and invasion of FZD2-mediated ESCC cells were independent from the canonical WNT signaling and indeed required STAT3 activation. FZD2 knockdown attenuated STAT3 activation at Tyr705 site, but not Ser727 site (Figure 3A). This result was confirmed by immunofluorescence staining for β-catenin in the KYSE150-shFZD2 and KYSE150-NC cells (Figure S1A). Moreover, the expressions of several key downstream targets of β-catenin, including transcriptional factor 1/7 (TCF1/7), CD44, and Met, were also unchanged in the KYSE150-shFZD2 cells compared with that in the KYSE150-NC cells (Figure S1B). In addition, only STAT3 Tyr705 phosphorylation, but not activated β-catenin, was observed to be increased in the FZD2-OE cells (Figure 3B).

It is well-known that TWIST1 and Slug are direct transcriptional targets of STAT3. These genes significantly enhance the EMT, migration and invasion of cancer cells, subsequently promoting metastasis (32). To determine whether FZD2-mediated STAT3 activation was associated with TWIST1/Slug-induced EMT, we compared the expression of TWIST1 and Slug between FZD2-OE or knockdown ESCC cells and their control cells, respectively. In the FZD2-knockdown ESCC cells (KYSE30 and KYSE150), the expression levels of TWIST1 and Slug were decreased (Figure 3A). In contrast, the expression levels of TWIST1 and Slug were increased in FZD2-OE KYSE410 cells compared with those in the control cells (Figure 3B). Based on these findings, FZD2-induced EMT required STAT3 activation in ESCC cells.

### FZD2 Promotes the Dissemination of ESCC Cells in a Mouse Model of Spontaneous Metastasis

**Figure 4:**
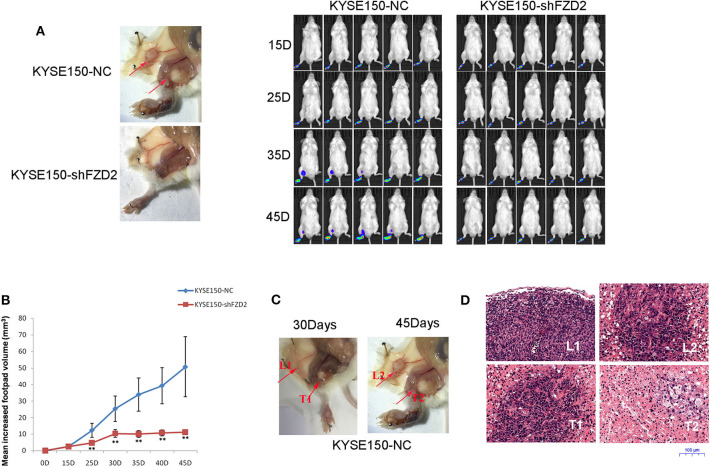
FZD2 promoted the metastasis of ESCC cells in a mouse model. **(A)** Representative images of swollen inguinal LNs and metastatic tumor cells (red arrows) in popliteal LNs of SCID mice following subcutaneous footpad injection of FZD2-knockdown KYSE150 cells and corresponding control cells were shown on the left. Live images of luciferase bioluminescence by Caliper IVIS Lumina II in mice were shown on the right. **(B)** The mean increased footpad volume following subcutaneous footpad injection of FZD2-knockdown KYSE150 cells and corresponding control cells on days 15, 25, 35, and 45 post-injection (**P < 0.01). **(C)** Representative images of swollen inguinal LNs and metastatic tumor cells on day 30 (L1 and T1, respectively) and day-45 (L2 and T2, respectively) post-injection. **(D)** Representative H&E staining of inguinal LNs and metastatic tumor cells on day 30 (L1 and T1, respectively) and day 45 (L2 and T2, respectively) post-injection (magnification 100 ×).

We established a spontaneous metastasis mouse model by injecting ESCC cells into the footpads of 4-weeks-old male SCID mice to further study the role of FZD2 in ESCC cell metastasis. Swollen popliteal LNs were observed in 80% (4/5) of the mice at day-45 post-injection of KYSE150-NC cells. In contrast, no swollen inguinal LNs or metastatic tumor cells were observed in the popliteal LNs from 5 mice injected with KYSE150-shFZD2 cells (Figure 4A). Additionally, the tumors composed of KYSE150-shFZD2 cells in the foot pads grew at a much slower rate than tumors of KYSE150-NC cells during the observation period (Figure 4B). We further analyzed the mice injected with KYSE150-NC cells and found that their inguinal LNs were swollen by day-30 post-injection (Figure 4C), and metastatic tumor cells had accumulated in their popliteal LNs at day-45 (Figure 4C). Lymphocytes in inguinal LNs were identified at day-30 post-injection, and these cells had disappeared by day-45, which were likely replaced by tumor cells in the KYSE150-NC group (Figure 4D). Thus, FZD2 silencing in ESCC cells significantly reduced LN metastasis.

### WNT2-Induced Migration and Invasion Is Dependent on FZD2 Overexpression

**Figure 5:**
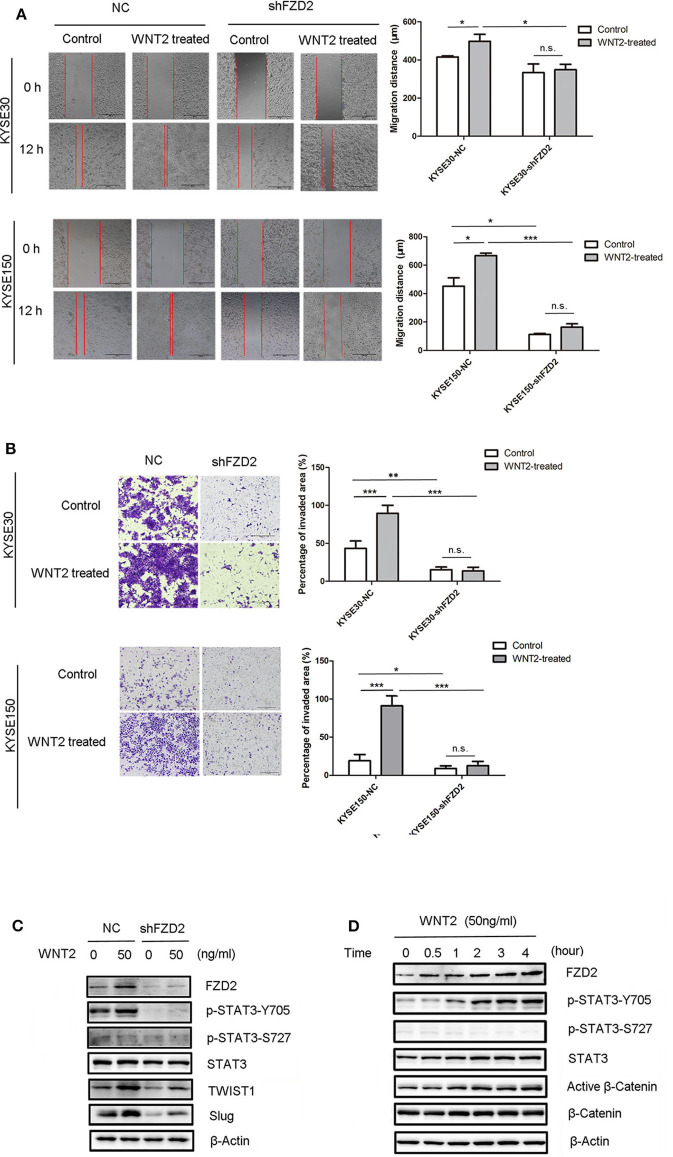
WNT2 induced the metastatic capabilities of ESCC cells via the FZD2/STAT3 signaling axis. **(A)** The migration distances of FZD2-knockdown ESCC cells and control cells with or without WNT2 treatment (50 ng/mL) were observed. Average migration distances are presented as the mean ± SD of triplicate experiments (*P < 0.05, ***P < 0.001 and n.s. non-significant; *t-*test). **(B)** The invasive capacity of FZD2-knockdown ESCC cells and corresponding control cells with or without WNT2 treatment (50 ng/mL). The percentage of invaded area were measured and are presented as the mean ± SD of triplicate wells (*P < 0.05, **P < 0.01, ***P < 0.001 and n.s. non-significant; *t*-test). **(C)** Western blot analysis of the levels of STAT3 signaling and its downstream mesenchymal markers TWIST1 and Slug in FZD2-knockdown KYSE150 cells and corresponding control cells with or without WNT2 treatment (50 ng/ml, 24 h). **(D)** Western blot analysis of FZD2, STAT3, p-STAT3-Y705, p-STAT3-S727, active β-catenin and β-catenin levels after WNT2 treatment (50 ng/mL) at 0, 0.5, 1, 2, 3, and 4 h.

A previous study reported that STAT3 interacts with FZD2 and plays a critical role in WNT5a/FZD2-mediated cancer cell metastasis (26). The secretion of WNT2 from cancer-associated fibroblasts is an important factor that promotes ESCC motility and invasiveness (22). A wound healing assay showed that the migration capacities of ESCC cells were enhanced in response to WNT2 treatment (50 ng/mL) for 12 h. However, the efficacy of WNT2-induced cell migration capacity was attenuated after the knockdown of FZD2 (Figure 5A). We also found that the invasive capacities of KYSE30 and KYSE150 cells were highly increased after the treatment with WNT2 protein (50 ng/mL) for 48 h. However, the WNT2-induced increase in the invasion of KYSE30 and KYSE150 cells was attenuated in FZD2-knockdown cells compared with control cells (Figure 5B). Moreover, the levels of STAT3 phosphorylation at Tyr705 and the STAT3-transcribed mesenchymal markers TWIST1 and Slug were substantially increased in the KYSE150 cells after WNT2 treatment, and these changes were attenuated by FZD2 knockdown (Figure 5C). TWIST1 and Slug were significantly down-regulated in the FZD2-deficient cells within 24 h after WNT2 treatment (50 ng/mL), compared to their expression in control cells. The FZD2 protein expression level was also upregulated after WNT2 treatment (Figure 5C). In addition, cell proliferation within 72h was examined but no significant changes were detected for the knockdown of FZD2 or WNT2 treatment in KYSE150 cells (Figure S2A). We also examined for the expression levels of biomarkers associated with cell survival (Mcl-1, Bcl-2, cIAP-2, and survivin) and proliferation marker cyclin D1, however, there was no significant difference when control or shFZD2 cells treated with WNT2 protein (50ng/ml, 24h) (Figure S2B).

Next, we examined the effect of WNT2 protein on FZD2 expression in ESCC cells. When the WNT2 protein (50 ng/mL) was added to the culture medium of ESCC cells, FZD2 levels were increased at 30 min post-treatment and remained at a high level throughout the 4-h examination. Interestingly, STAT3 phosphorylation at Tyr705 was progressively increased in a time-dependent manner by WNT2 treatment, but the Ser727 site remained unchanged (Figure 5D). Because the p-STAT3-Y705 level was increased after FZD2 aggregated, we inferred that STAT3 activation may be influenced by FZD2. In contrast, in the KYSE150-shFZD2 cells, WNT2 induced lower increased levels of FZD2 expression and STAT3-TWIST1/Slug signaling activation compared with the control cells (Figure 5C).

Thus, WNT2-induced migration and invasion in ESCC cells was dependent on FZD2 expression and associated with STAT3 signaling.

### WNT2-Induced FZD2 Expression Depends on the Inhibition of Ubiquitination-Mediated Protein Degradation

**Figure 6:**
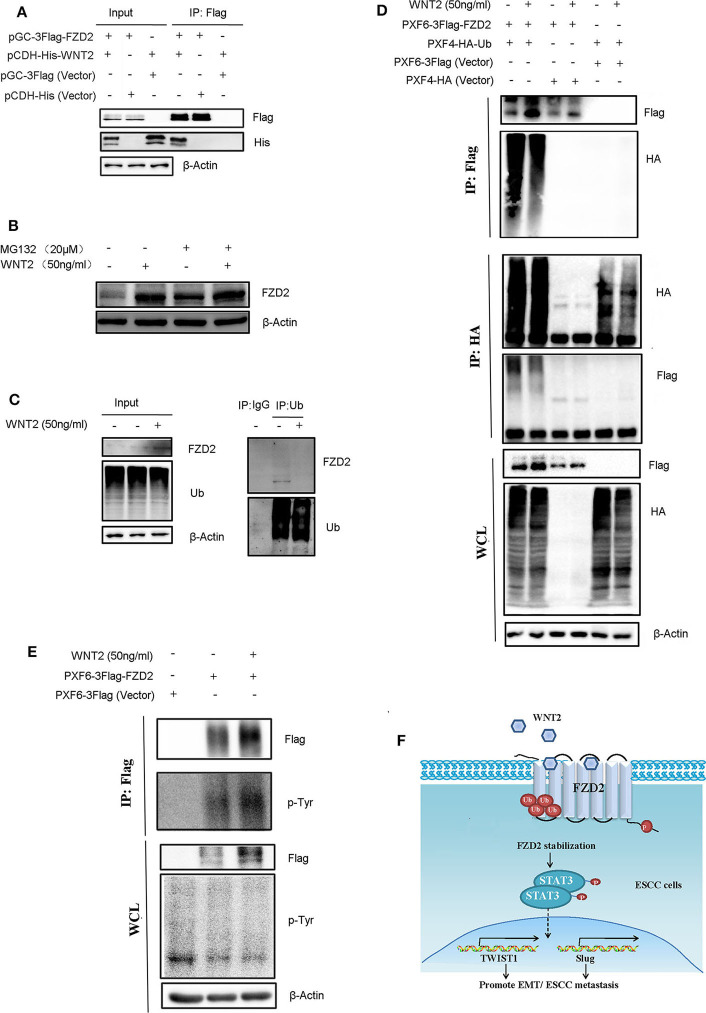
WNT2 ligand stabilized and phosphorylated the FZD2 receptor. **(A)** WNT2 interacts with FZD2 in the ESCC cells. The lentiviral vectors overexpressed the Flag-tagged FZD2, the His-tagged WNT2 or the empty lentiviral vectors for controsl were stably infected KYSE150 cells according to the labels. The whole cell lysates (WCL, input) were detected using western blot analysis (3 panels on the left). The Flag-tagged FZD2 was immunoprecipitated with Flag-M2 beads, and immunoblotting for Flag and His was performed (3 panels on the right). **(B)** Western blot analysis for FZD2 protein levels of control and under WNT2 (50 ng/ml) exposure cells after treatment with MG132 (20 μM) for 6 h or not. **(C)** WNT2 reduced FZD2 ubiquitination. Total tissue lysates extracted from KYSE150 cells were immunoprecipitated by Ub antibody and probed with indicated antibodies. Non-specific mouse IgG was used as control. **(D)** Flag-tagged FZD2 and HA-tagged Ub were transfected into HEK293T cells for 48 h and then treated with or without WNT2 (50 ng/mL, 4 h). Empty vectors were used as controls. Western blot analysis showed exogenous FZD2 and Ub expression (WCL, lower panel). Flag-tagged FZD2 was immunoprecipitated with anti-Flag M2 beads (IP: Flag, upper panel); total ubiquitinated proteins were immunoprecipitated with the anti-HA antibody (IP: HA, middle panel); and immunoblotting was performed with the indicated antibodies. **(E)** WNT2 increased tyrosine phosphorylation of FZD2. Flag-tagged FZD2 plasmid and the empty vector plasmid were transfected into the HEK293T cells for 48 h, and then, the cells were treated with or without WNT2 (50 ng/mL, 4 h). Western blot analysis showed the exogenous FZD2 levels and total phosphotyrosine levels in all lines (WCL, lower panel). Flag-tagged FZD2 was immunoprecipitated and immunoblotting was performed with total phosphotyrosine or Flag antibody (IP: Flag, upper panel). **(F)** Proposed mechanism of WNT2/FZD2 signaling pathway in ESCC. WNT2 can bind to FZD2 receptor. WNT2-induced ESCC cell migration and invasion is dependent on FZD2 overexpression by inhibiting its ubiquitination-mediated protein degradation. FZD2 promotes metastatic capabilities of human ESCC cells by activating STAT3 signaling.

To identify the interaction between WNT2 and FZD2, we packaged overexpressed lentiviruses for WNT2 and FZD2 and infected the KYSE150 cells for co-immunoprecipitation assays to test the interaction between WNT2 and FZD2 proteins. The results showed that WNT2 and FZD2 interacted with each other (Figure 6A). Since WNT2 increased the expression level of endogenous FZD2 (Figure 5D), we further investigated whether the FZD2 protein level was affected by ubiquitination and proteasome-mediated degradation in response to WNT2 treatment. The result showed that the FZD2 expression levels can be increased after WNT2 or MG132 treatment, respectively (lane 1 vs. lane 2; lane 3 vs. lane 4; lane 1 vs. lane 3). However, there is no significantly additive increase of FZD2 after WNT2 and MG132 co-treatment, which may attribute to the deubiquitination of FZD2 (lane 2 vs. lane 4) (Figure 6B). Immunoprecipitation for ubiquitin in KYSE150 cells revealed that the ubiquitination of FZD2 was reduced when WNT2 protein treated (Figure 6C). When the Flag-tagged FZD2 expression construct (Flag-FZD2) and an HA-tagged ubiquitin (HA-Ub) plasmid were co-transfected into HEK293T cells with or without WNT2 treatment, robust expression and ubiquitination of FZD2 were detected in the whole cell lysates (WCLs). Subsequent IP assays with an anti-Flag antibody showed that the level of the slow migrating smear of HA-Ub was attenuated in response to WNT2 treatment. The decrease in the smear indicated that the binding of ubiquitin molecules to the FZD2 protein was reduced in response to WNT2 exposure (Figure 6D, IP: Flag, upper panel). Meanwhile, immunoprecipitation with anti-HA antibody revealed that the smear resulting from ubiquitinated Flag-FZD2 was also reduced in response to WNT2 treatment (Figure 6D, IP: HA, middle panel). Additionally, western blot assays revealed an increase in the endogenous and exogenous FZD2 expression after WNT2 treatment (Figures 6C,D).

Next, the tyrosine phosphorylation of FZD2 was detected by immunoblotting with anti-phosphotyrosine antibody (p-Tyr) in immunoprecipitates with Flag-FZD2 pulldown. We also found that the FZD2 level increased in WCLs and immunoprecipitated samples with the Flag antibody were treated with WNT2. Additionally, the tyrosine phosphorylation of FZD2 was also increased after WNT2 treatment, as detected using a p-Tyr antibody Figure 6E). Therefore, WNT2 likely binds to FZD2, inhibiting its ubiquitination and degradation, and inducing its tyrosine phosphorylation.

Overall, these data indicate that WNT2-induced ESCC cell metastasis is dependent on FZD2 overexpression by inhibiting its ubiquitination-mediated protein degradation, leading to the activation of STAT3 signaling and initiation of EMT process (Figure 6F).

## Discussion

Despite extensive research in the diagnosis and treatment of ESCC, the 5-year survival rate of patients after curative surgery remains low (only 20–30%). The poor outcomes mainly result from the occurrence of tumor metastasis and recurrence (33, 34). However, the molecular mechanisms underlying ESCC metastasis are not yet completely understood.

We previously showed that CAFs-secreted WNT2 is a metastasis-promoting WNT ligand in ESCC (22). Here we screened the potential binding receptors of WNT2 and further investigated the significance of FZD2, in the pathogenesis of ESCC. Despite the tumor-promoting function of WNT5a/FZD2 pathway was found in previous study, *WNT5A* is frequently silenced by promoter CpG methylation in ESCC (26, 35), indicating that WNT5A may not be a critical ligand binding to FZD2 in ESCC progression. We performed a detailed investigation to determine the association between WNT2 and FZD2 in ESCC cells and in clinical specimens.

The canonical and non-canonical WNT pathway plays important roles in tumourigenesis and tumor metastasis (36–38). Previous studies have shown that FZD2 promotes cancer development by activating both the canonical and non-canonical WNT pathways (24–26). Here, FZD2, which induced STAT3 activation, induced the migration and invasion of ESCC cancer cells, and a high level of FZD2 expression predicted a poor prognosis for patients with ESCC. Therefore, FZD2 is an oncogene that is involved in the maintenance of the mesenchymal phenotype and the metastasis of ESCC cells.

Accumulated evidence has revealed the importance of LN metastasis in the progression of many types of tumors (39–41). Regional LN metastasis is usually considered to be an early step for ESCC dissemination and progression, leading to poor prognoses (39, 42). In this study, we found that inhibiting FZD2 expression in ESCC could significantly reduce LN metastasis, which may serve as a potential therapeutic target for the treatment of ESCC patients.

STAT3 plays important roles in the progression of various cancers by regulating the proliferation, invasion, angiogenesis and immune surveillance evasion (42, 43). The Tyr705 phosphorylation site in STAT3 translocates into the nucleus and binds to the specific promoter sequences of various EMT master transcription factors, such as TWIST and Slug (32, 44). Constitutive STAT3 activation is associated with the progression of ESCC and poor prognosis (45). As shown in the present study, FZD2 knockdown reduced STAT3 phosphorylation on Tyr705 in ESCC cells, consistent with the findings by Gujral et al. (26). Furthermore, we demonstrate that FZD2 induces EMT and metastasis in ESCC cells via the STAT3/TWIST1 and STAT3/Slug pathways.

The tumor microenvironment is crucial for the development of cancer (46, 47). Cancer-associated fibroblast-secreted WNT2 acts as a growth- and invasion-promoting factor in ESCC cells (22). We found that *FZD2* mRNA was co-expressed with *WNT2* mRNA in ESCC, suggesting a positive correlation between FZD2 and WNT2 levels. In the present study, FZD2 directly interacted with WNT2, thereby activating the STAT3 signaling pathway in ESCC cells. We demonstrated, for the first time, that *WNT2* and *FZD2* were both positively correlated with mesenchymal cell markers but negatively correlated with epithelial cell markers (TCGA-ESCC database analysis). The WNT2 protein not only induced ESCC metastasis as we previously reported (22), but also activated STAT3 at Tyr 705 site by binding to, stabilizing and phosphorylating FZD2. To the best of our knowledge, this report is the first to describe this interaction between WNT2 and FZD2. However, alternative mechanisms for WNT2-induced STAT3 activation are worthy of further investigations.

In conclusion, we demonstrated a novel molecular mechanism underlying the effects of WNT2-FZD2 on ESCC metastasis. Moreover, strategies that target the WNT2/FZD2/STAT3 signaling axis might be developed to treat patients with ESCC.

## Data Availability Statement

The original contributions presented in the study are included in the article/Supplementary Material, further inquiries can be directed to the corresponding author/s.

## Ethics Statement

The studies involving human participants were reviewed and approved by the Institutional Review Board and Ethics Committee of the First Affiliated Hospital of Zhejiang University. The patients/participants provided their written informed consent to participate in this study. The animal study was reviewed and approved by the Experimental Animals Ethics at the Zhejiang Chinese Medical University. Written informed consent was obtained from the individual(s) for the publication of any potentially identifiable images or data included in this article.

## Author Contributions

YF, ZC, LF, and TC: conceived the project, designed the experiments, and wrote the manuscript. YM, XJ, YC, and TC: analyzed the public databases and wrote the manuscript. YF, QZ, and XC: performed and interpreted the majority of the experiments. PL, TH, JY, CD, XD, TS, XW, YY, and WJ: performed experiments. XC, BL, and CZ: collected the clinical samples and analyzed the data. ZC, LF, and TC: supervised the project. All authors read and approved the final manuscript.

## Conflict of Interest

The authors declare that the research was conducted in the absence of any commercial or financial relationships that could be construed as a potential conflict of interest.
