# Supplementary material for: WNT2-Mediated FZD2 Stabilization Regulates Esophageal Cancer Metastasis via STAT3 Signaling
Source: Front Oncol. 2020 Jul 16;10:1168. doi: 10.3389/fonc.2020.01168 (PMC7379028; doi:10.3389/fonc.2020.01168)
Supplement: Supplementary file 1 [file Table_1.docx]

**TABLE S1**

Correlation between FZD2 expression and clinic pathological features.

|  | | | **Sex** | **Age** | **Tumor size** | **Pathological grade** | **T** | **N** | **Clinical stage** |
| --- | --- | --- | --- | --- | --- | --- | --- | --- | --- |
| Spearman's rho | FZD2 grouping  in ESCC | Correlation | -0.003 | 0.127 | -0.215* | 0.160 | 0.160 | 0.047 | 0.044 |
|  |  | Coefficient | 3 | 7 | - | - | 0 | 7 | - |
|  |  | *P*-value | 0.977 | 0.207 | 0.048 | 0.111 | 0.113 | 0.654 | 0.673 |
|  |  | N | 100 | 100 | 85 | 100 | 99 | 96 | 95 |
|  | FZD2 score  in ESCC | Correlation | 0.056 | 0.118 | -0.148 | 0.244* | 0.070 | 0.083 | 0.242* |
|  |  | Coefficient | 6 | 8 | - | - | 0 | 3 | - |
|  |  | *P*-value | 0.581 | 0.244 | 0.876 | 0.014 | 0.494 | 0.419 | 0.015 |
|  |  | N | 100 | 100 | 85 | 100 | 99 | 96 | 100 |

**Score and grouping:**

Score of staining intensity: 0= Score 0, 1= Score 1, 2= Score 2, 3= Score 3;

Grouping: total score<2 and total score≥2 refer to the “FZD2 low expression group” and “FZD2 high expression group”, respectively.

***:** *P*<0.05
