# Supplementary material for: WNT2-Mediated FZD2 Stabilization Regulates Esophageal Cancer Metastasis via STAT3 Signaling
Source: Front Oncol. 2020 Jul 16;10:1168. doi: 10.3389/fonc.2020.01168 (PMC7379028; doi:10.3389/fonc.2020.01168)
Supplement: Supplementary file 2 [file Data_Sheet_1.docx]

**Supplementary figure legends**

**FIGURE S1. WNT2 and FZD2 had no effect on the proliferation of ESCC cells. (A)** Proliferation rates of KYSE150 FZD2-knockdown (shFZD2) and control (NC) cells with or without WNT2 (50ng/mL) treatment within 72h were determined using CCK-8 assays. There is no significant difference on cell proliferation by two-way ANOVA analysis (F=2.91, P=0.101). **(B)** Cell survival and proliferation related downstream targets of STAT3, including Mcl-1, Bcl-2, cIAP-2, surviving and cyclin D1 were detected using western blot analysis.

**FIGURE S2. FZD2 knockdown did not influence canonical β-catenin signalling and downstream targets.** **(A)** Representative images showed the expression of β-catenin in FZD2-knockdown cells (shFZD2) and corresponding control cells (NC). **(B)** Downstream targets of β-catenin, including CD44, Met and TCF1/TCF7, were detected using western blot analysis.
