# Supplementary figures and images for: WNT2-Mediated FZD2 Stabilization Regulates Esophageal Cancer Metastasis via STAT3 Signaling
Source: Front Oncol. 2020 Jul 16;10:1168. doi: 10.3389/fonc.2020.01168 (PMC7379028; doi:10.3389/fonc.2020.01168)

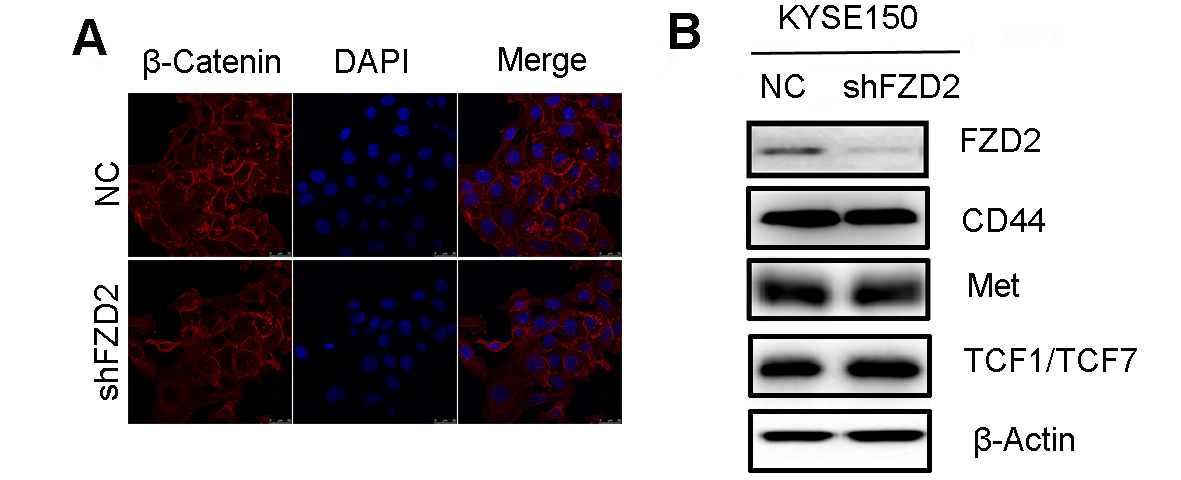

Supplement: Supplementary file 3 [file Image_1.TIF]

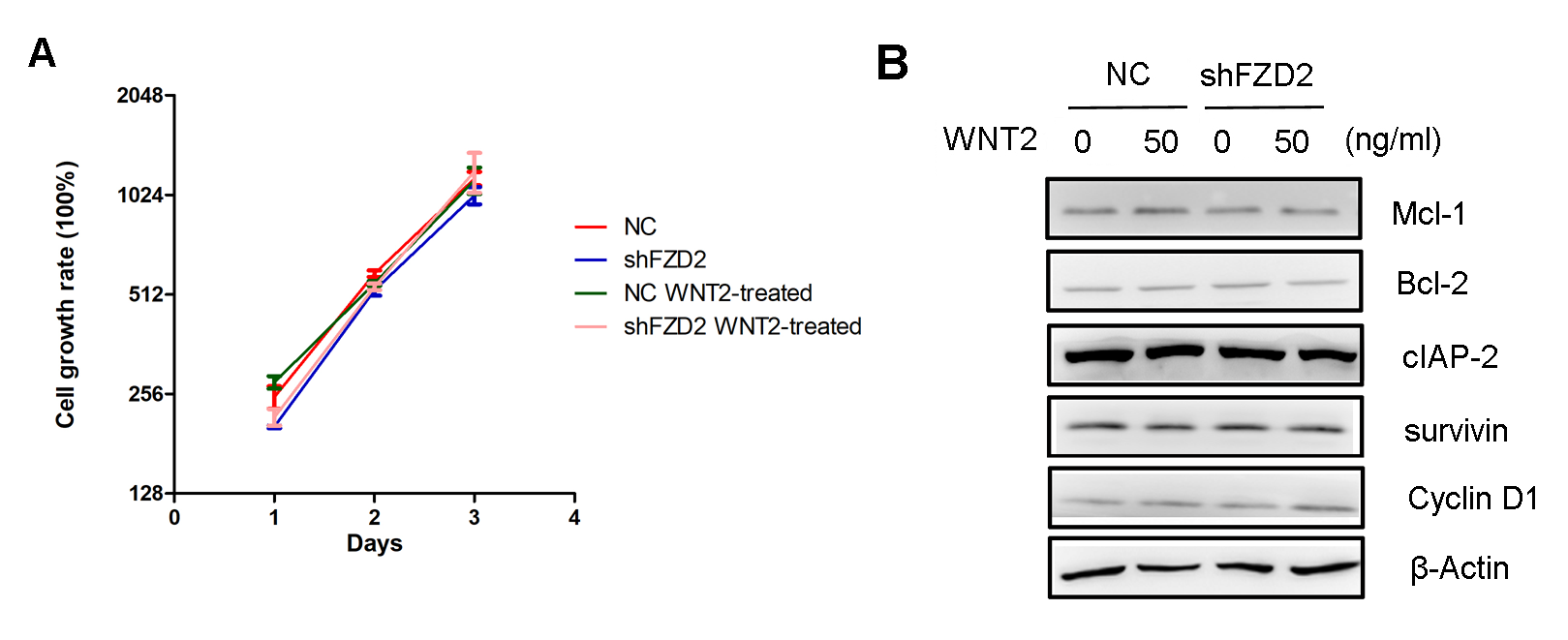

Supplement: Supplementary file 4 [file Image_2.TIF]
